# “Hanging on by a Thread”: The Lived Experience of Parents of Children with Medical Complexity

**DOI:** 10.3390/children11101258

**Published:** 2024-10-18

**Authors:** Courtney Holmes, Waganesh Zeleke, Shruti Sampath, Tiffany Kimbrough

**Affiliations:** 1 Department of Rehabilitation Counseling, Virginia Commonwealth University, Richmond, VA 23298, USA; zelekew@vcu.edu (W.Z.);; 2 Virginia Commonwealth University Health System, Children’s Hospital of Richmond, Richmond, VA 23219, USA; tiffany.kimbrough@vcuhealth.org

**Keywords:** trauma, resilience, medical complexity, parenting, complex care

## Abstract

Background: Families with children with medical complexity endure high levels of chronic and toxic stress, impacting the overall health and wellbeing of all family members and the system as a whole. The purpose of this study was to examine the lived experiences of parents/caregivers with children with medical complexity. Methods: The lived experiences of 15 parents of children with complex medical needs were explored using focus group discussions. Focus group discussions were recorded, transcribed, and analyzed using van Manen’s 6-step process for hermeneutic phenomenology. Aligned with the research questions, the analysis draws on van Manen’s four lived existential analytical categories. Results: Themes include: (1) lived body, the psychological and physiological effect; (2) lived time and space, the immediate impact on the there, now, and then; (3) lived relationships, social life and family relational effect; (4) risk factors; and (5) protective factors. Conclusion: Caregivers of children with medical complexity have a unique experience of trauma and resilience. This study will inform interdisciplinary medical providers about the mental health and resiliency experiences of this population to support more effective healthcare practices.

## 1. Introduction

Families with children with medical complexity face health inequities and untenable levels of reported physical and mental health problems. Family members of children with medical complexity endorse substantial unmet mental and behavioral healthcare needs [1]. Approximately 19% of US children have special healthcare needs, defined by the Maternal and Child Health Bureau as having or being at increased risk for a chronic physical, developmental, behavioral, or emotional condition and requiring health and related services beyond that required by children generally [2,3,4]. An estimated 3 million US children are considered to have medical complexity [3]. This group of children is the most medically fragile and has the most intensive healthcare needs [2,3,5], intensive utilization of medical providers (either hospital or outpatient), need for care coordination, reliance on assistive technology, polypharmacy, and ongoing home care to maintain an essential quality of life [2,6,7]. Worldwide estimates of children with medical complexity are not easily accessible, but comprehensive care programs continue to be established internationally (Harvey). In the US, children with medical complexity (CMC) account for less than 2% of the pediatric population, while accounting for 30% of healthcare spending nationally [8,9]. Researchers have suggested that in the US, medical complexity is itself a social determinant of health, severely impacting a wide range of family health outcomes [8,10].

CMC have disease processes and chronic conditions that impact their overall functioning, abilities, and life expectancy. They require ongoing treatment from multiple specialists, medications, specialized equipment, therapy services, school accommodations, and other healthcare aids. Despite continuous healthcare provider involvement, these children and their families often suffer from significant and largely unmet mental health needs [7,11]. The term “toxic stress” refers to a set of biological changes that occur at internal and external physical levels (e.g., molecular, cellular, and behavioral) when there is sustained or significant adversity without mitigating social/emotional buffers [12]. Ongoing and unresolved trauma, financial burdens, isolation, and overwhelming caregiver stress all negatively impact family well-being. Families with CMC are at significant risk of poor mental health and family functioning, indicating a relationship between lower levels of family functioning and increasingly intensive medical requirements [7,8,11]. Despite sustaining critical levels of physical, mental, and social burdens, parents often do not seek support [1,13].

Parents and caregivers of CMC face untenable levels of stress [2,6,11,14] starting with the initial diagnosis, continuing with unpredictable health outcomes with intermittent health crises, financial concerns, and ongoing interactions with medical professionals [15]. Caregiver burden and strain are well documented in parents of children with medical complexity [11,16] as negative impacts include physical, psychological, and financial decline [1,11]. Parent caregivers consistently report elevated levels of depression, anxiety, fatigue, distress, poor physical health, cardiovascular risks, headaches, decreased cognitive function, isolation, emotional distress, and other mental health complaints [14,17,18,19]. Moreover, family members report that despite ongoing interfacing with the healthcare system, mental and behavioral healthcare needs remain unmet [8,19,20]. Over 80% of families in one sample reported challenges with family daily activities, including problems with family relationships, lack of communication, and increased stress and conflict [1,21,22]. The mental health burden for children with medical complexity and their families became exacerbated by the COVID-19 pandemic [23,24,25]. Many families lost their ability to meet home nursing hours, faced equipment and supply challenges, and lost their critical social network during extended isolation and quarantine periods [24,25]. Financial burdens intensified due to job insecurity while out-of-pocket costs increased. Social isolation, caregiver mood symptoms, and food insecurity increased during the pandemic in a pediatric family sample [23].

Parents are often likely to encounter some form of medical trauma during their children’s lives, putting them at a higher risk for developing conditions like Post-Traumatic Stress Disorder (PTSD), depression, anxiety, and complicated grief [25,26]. The psychological effects can include mourning the loss of hopes for their child, witnessing their child in pain, experiencing marital tensions, chronic fatigue, social isolation, and feeling unsupported by family and friends [1,5]. Medical trauma can stem from parents perceiving their child as enduring significant pain, fearing for their child’s life, or worrying about the possibility of a serious medical diagnosis [27,28,29] impacting increased rates of clinically significant depression, anxiety, and suicidality [13,27,28]. Traumatic experiences can lead to unhealthy coping mechanisms and various negative health outcomes, including heightened anxiety and depression, increased substance use, cardiovascular problems, chronic pain, and other physical illnesses [7]. Furthermore, results do not differ significantly, as international publications agree, “that poor care for this group has negative implications for CMC and their families, but also for society as a whole due to inefficiencies in healthcare delivery and spiraling healthcare costs” [10] (p. 9).

While most people experience at least one traumatic event in their lifetime, this type of chronic and unrelenting traumatic experience related to a child’s health is not well understood. Furthermore, the mental health of parents of CMC is poorly understood, yet parent wellness drives child and family health outcomes and quality of life [7]. The literature addresses parents’ experiences with children in the Neonatal Intensive Care Unit [30]; however, it does not follow parent experiences as children are released with ongoing chronic and intensive healthcare needs. Parents of CMC experience medical trauma consistently as their children are frequently in and out of hospital care, need lifesaving interventions, and sustain chronic illness. This level of sustained emotional upset is likely to cause toxic stress in this group of parent-caregivers. In any medical trauma, the ubiquitous experience of the parent and its effect on the parent’s well-being should be considered. However, woefully, the lived experience of this group is underrepresented in the research, as is a strength-based focus on resiliency factors. A more thorough understanding of parent caregivers’ experiences will assist medical providers in assessing parent well-being and increasing the effective delivery of trauma-informed care in healthcare settings. This article is one small part of a larger need for increasing awareness for the longitudinal traumas experienced by caregivers of CMC. Increased awareness allows for improved empathy and trauma-informed care in the healthcare setting as we approach care delivery models for patients and families. Healthcare providers for families with CMC are in a unique position to offer support to families; thus, insight into the lived experiences is imperative [7,31,32].

### Purpose

This study aims to understand better parents’ experiences of children with medical complexity as they relate to trauma, stress, and resilience. The study used a phenomenological qualitative research approach [33,34] and sought answers to the following questions:

(1) What is the lived experience of parents and caregivers of CMC?
(2) What risk factors exist in the families and healthcare service context of parents of CMC?
(3) What protective factors need to be constructed for parents and caregivers of CMC?

## 2. Materials and Methods

This qualitative study is guided by the phenomenological approach using hermeneutic interpretive phenomenology. As a philosophical and methodological approach, hermeneutic interpretive phenomenology focuses on producing reconstructed understandings [33,34]. As a research method, it involves the hermeneutic circle of grasping the meaning of the phenomenon by understanding the part and the whole [33]. In this method, emphasis is placed upon seeing all individuals in their context and knowledge that each person uniquely perceives the world. The Virginia Commonwealth University’s Institute of Research Board approved this study before recruitment and data collection.

### 2.1. Methodological Context of the Current Study

Little is known about the unique experiences of parents with CMC as it pertains to trauma and resilience. In the United States, parents may face many accessibility barriers to mental healthcare. If mental healthcare is sought, it is unlikely that the provider has insight into the unique needs of this population. This study sought to explore the parenting experiences of this population to better understand the impact of trauma and their perception of resilience within their family context.

### 2.2. Informants

The Complex Care Clinic at Children’s Hospital of Richmond enrolled over 300 families during this study; all eligible families were the target population. Eligibility included being over 18 years of age, having a child diagnosed with a complex medical condition, and speaking English. Recruitment methods were collaborative between the primary researcher and the medical director of the Complex Care Clinic. The primary researcher sent emails to all parents enrolled in the clinic. Families involved in a community-based non-profit serving medically complex kids were contacted via email by the non-profit director to supplement the recruiting strategies of the target population. Interested parents filled out a REDCap form and were contacted by the primary researcher with study information [35,36]. Twenty-one informants expressed interest, and 15 attended the focus group discussion. Five focus groups were held for 45–60 min via Zoom with 3–4 members per group. Participants were compensated with a $30 Walmart gift card for participation.

### 2.3. Instrument and Data Collection Procedure

First, Institutional Review Board approval was granted in the fall of 2023. This project was funded through an internal college initiative at the researchers’ institution. Recruitment was completed through email. Interested parents contacted the research assistant and consented to the project, and focus groups were scheduled between October 2023 and January 2024. Focus group discussions (FGD) were held with parents of children with complex medical conditions. Focus groups are a typical group interview method for thematic analysis and a suggested methodology for reducing barriers to participation [37]. The semi-structured questions in the FGDs include: What are your experiences as a parent or caregiver of a child with complex medical needs? Specifically, what is your experience of stress, and how does it impact your parenting? How have your relationships been affected through this experience of parenting? How has chronic stress impacted your body, physical, and mental health? What feels like the most important thing that shapes your experience as a parent? What is your experience with resilience as a family, and what do you love about your family? The primary researcher and a research assistant conducted the semi-structured focus group discussions to ensure consistency and allow one person to observe the group dynamics and discussion.

### 2.4. Analysis

Once the focus group discussions had been transcribed and uploaded to the “Delve, a cloud-based CAQDAS tool for coding in qualitative research [38] for coding and analysis, three individuals, including the graduate assistant, principal investigator, and faculty collaborator, thoroughly read and reread the transcripts. The team organized meetings to discuss and establish a shared understanding of the informants’ responses, using debrief codes and trigger statements, which succinctly summarized or explained issues [33].

The transcriptions were analyzed using van Manen’s 6-step process for hermeneutic phenomenology, which comprises the following stages: (1) focusing on the nature of lived experience, (2) investigating experiences as they are lived, (3) identifying the essential themes that characterize the phenomenon, (4) describing the phenomenon in writing and rewriting, (5) sustaining a strong and focused relationship with the phenomenon, and (6) balancing the research context by considering both the parts and the whole [33,34]. The coding process involved each author reading the transcripts independently and highlighting specific sections that pertained to the lived experiences of parents and caregivers. These sections were labeled with codes and subsequently combined into concepts using the Delve software [38]. To reflect on the meaning of these concepts and identify the essential themes that characterize the experience of parents related to dealing with their children’s medically complex needs, the principal investigator and a faculty collaborator developed 39 codes under six categories separately and held four meetings to discuss their thoughts on the definitions crafted for each concept and reread the relevant texts. The third coder then compared notes with the other two to ensure that as much richness from the text as possible had been captured. Then, a unified list of concepts and definitions that describe the phenomena of parenting a child with complex needs was created, and five major themes emerged from the six categories. This process was cyclical, with the authors often contrasting concepts with the structure of the research aim and vice versa.

### 2.5. Trustworthiness

The trustworthiness of this study is established through several rigorous methods, including maintaining an audit trail, incorporating direct participants’ quotes, and employing member checking. The research team meticulously documented each data analysis step to mitigate bias and ensure transparency. The principal investigator initiated the process by collaborating with graduate research assistant students to discuss the quality of the focus group discussion process. This was followed by two preparatory meetings before conducting the focus group discussion. A total of five group discussions were held, with one researcher facilitating the discussion and one observer. After the focus group discussions, the principal investigator and the research team convened four additional meetings to discuss the process and the next steps of the coding and theme development. During these meetings, the team engaged in thorough discussion and cross-checked each other’s interpretation of the data to enhance the accuracy and reliability of the findings.

The study’s results are further substantiated by numerous direct quotes from participants, providing a rich, authentic representation of their experience. To ensure the validity of the analysis, participants were also involved in the data analysis process through member checking, where they reviewed and confirmed that the derived themes accurately reflected their experience. This participant feedback validated the findings, reinforcing the study’s credibility.

## 3. Results and Findings

This study draws empirical findings from focus group discussions of 15 parents of CMC about their lived experience dealing with their child’s complex medical needs. The demographic makeup of the sample is shown in Table 1. While specific diagnostic information was not collected, each participant’s child met the criteria for receiving treatment in the complex care clinic, meaning they received treatment from five or more specialists.

The following section presented the data analysis using a phenomenological inquiry. The data analysis is structured according to the research guiding questions:

(1) What are the lived existential experiences of parents and caregivers of CMC and the four associated subsidiary questions: What are the lived existential dimensions of the body, time, space, and others?
(2) What risk factors exist within the families and healthcare service context of parents of CMC?
(3) What protective factors need to be constructed for parents and caregivers of CMC?

Aligned with the research questions, the analysis draws on van Manen’s four lived existential analytical categories (lived space, lived body, lived time, and lived others) alongside Bronfenbrenner’s bioecological model of risk and protective factors to conduct a cross-case thematic analysis (see Table 2).

### 3.1. Lived Existential Experience of Parents and Caregivers

#### 3.1.1. Lived Body: The Psychological and Physiological Impact

Participants in the study reported significant changes in their physical and psychological well-being following the diagnosis of their child’s medical complexity. These changes included weight gain, sleep disturbance, increased irritability, trauma symptoms, stress, and exhaustion. One participant noted, “Well, when my daughter was first hospitalized, I gained a significant amount of weight from both, I think, stress and living in a hospital for several months, and commuting to the hospital immediately after work,” and “Physically, there were times where she spent months in the hospital, so I slept in the hospital and only ate hospital food and stressed-ate and gained a ton of weight really fast”.

Chronic and toxic stress was a shared experience, with one participant expressing, “You’re just a pin drop away from losing your mind where the stress gets to an absolute peak, then it just lives there”. Another shared, “I brought myself to the emergency room when my daughter was in the ICU, because I swore I was having a heart attack.” and “I feel like I’m at least ten years older than my chronological age from a physical standpoint”. Another participant highlighted the experience of trauma: “But if you’re coming across a person whose child is medically complex, that parent has medical trauma, end of story, period”.

The shared participants’ narratives highlighted psychological struggles, such as complete and chronic exhaustion, with no relief. Participants shared, “I know with me, it’s just exhaustion. Short temper and exhaustion have been my result that I have noticed. I look for ways to mitigate it, but it seems like it is always there…”; “Short temper and exhaustion have been my results that I have noticed”. Another shared, “I have terrible sleep because of the schedule, and I can’t nap because my son requires endless care…he doesn’t nap, and he only sleeps about nine hours a night”.

The psychological toll also manifested as a profound sense of isolation, seeming from the feeling that others could not understand their experience. One participant shared, “Just that feeling of nobody understands this, nobody gets this, and who do I turn to?” Another shared, “But I’m in a situation where I feel like if I share, they won’t fully understand anyway”. This sense of isolation often extended to interactions with friends and family, leading to a feeling of disconnection even when physically surrounded by people. One participant shared, “…it often feels like most other parents don’t understand that, and they can’t necessarily relate to that, so I think that compounds the stress a little bit, to feel like it’s a little isolating knowing that other people don’t really get what you’re going through”.

Dealing with ambiguity was another experience shared by participants. One participant shared, “To think that every single time your child goes to the hospital, you are not sure if they are going to come home, it just takes a toll on you”. To illustrate daily life and the ambiguity surrounding making plans, one participant stated, “You can’t be rigid about your plans. If you are, you end up frustrated. The same thing with the other family members, everybody has to be able to be flexible and willing to change and willing to roll with things or people will just start snapping at each other”. To further highlight the ambiguity of daily life, one participant agreed, “The plans change immediately”.

Grief was a shared experience among participants that impacted physical and psychological functioning. Grief was expressed through a myriad of lived experiences, including individual loss of a child, first- and second-hand exposure to extreme medical situations, preparatory grief, and ambiguous grief. To illustrate this experience, one participant stated, “You can see the other babies right across from you, so I still feel like I have trauma from seeing so many people go through so many terrible losses and so many babies just struggle, and then our son”. Another shared, “You can’t sleep there, but I was there every minute I could be, and you just see lots of babies who don’t make it”. Related to ambiguous grief, one participant shared, “And so periodically there just are those dips of feeling that pretty profound grief over what could have/should of/would of”. Another shared, “I don’t sit in it, but there is mourning and grief for the more typical imagined life”. Finally, one shared, “We love our children and would slay dragons for them and die for them, but this is not what we imagined parenthood was going to be like, and taking us out of it, nor is this what we wanted for our child”.

#### 3.1.2. Lived Time and Space: Impact on Daily Life

The diagnosis of a child with complete medical needs led to profound changes in participants’ life experiences of time and space. One participant described feeling “hanging by a thread,” reflecting chronic physical and mental exhaustion. The constant anticipation of medical emergencies created a persistent sense of anxiety, as one participant noted, “No matter how much joy there is in life, you do have this thing in the back of your mind all the time about the what ifs and what’s going to happen and how different your life is”.

The limitations on physical space were also significant. Participants often felt confined to their homes and unable to engage in social activities or travel. One participant highlighted, “For the last ten years, it’s been impossible to go out and do things. You just can’t go out and do things with people”. The necessity to relocate to be closer to healthcare facilities also emerged, with one participant stating, “It was hard going from city to city”. Another said, “I had to switch my daughter to the school by the hospital”.

The inability to take breaks or vacations exacerbated the feeling of being trapped, with caregiving responsibilities consuming all available time. One participant explained, “Getting away is not impossible, but it is incredibly complex and incredibly difficult”. One participant shared, “I have one place, one place in the entire world that I can go to for a vacation”. Participants discussed the all-consuming healthcare and management responsibilities that become part of the job of the parent to a medically complex child. One participant shared, “It takes me and my parents-in-law and our full-time caregiver and his full-time night-time caregiver, and then he has full-time staff at school, as well. The eight of us, or whatever, get it done. It’s a lot just like a regular job”. Another shared, “I feel like I often can’t take a break.” and “There’s no time for respite basically”. Finally, participants discussed how the chronic responsibilities impact experiencing joy through parenting: “It is hard to… just relax and enjoy being a parent”.

#### 3.1.3. Lived Others: Social and Family Relationship

Participants shared the critical importance of support through family, friends, and healthcare providers. Relationships for parents with CMC are multifaceted. On one hand, perceived disconnection from others who do not share the same experiences is pervasive (discussed in theme one through experiences of isolation). However, despite these experiences, participants described immense gratitude and appreciation for connections with supportive people who did understand and attempted to. For example, “…just the people who do understand really showing up for us has been huge for me”. Another shared, “I’m so thankful for my baby, and I’m also thankful for the support that I have around me, and that helps to relieve the stress like I have some other NICU moms I met when my sole community was a lot of the nurses from the NICU”. One participant stated, “I just think leaning on the support that we do have, that’s been huge for us”. Another shared, “My parents and my husband’s parents are really supportive”. Related to the importance of healthcare staff, participants stated, “To have a team helping support with all that transition was incredibly helpful”. Concerning the meaning of support, participants discussed their transition to accepting help and letting others in. For example, one participant stated, “But this year, I’ve let our nanny drive him to therapy and be responsible for that, and that has been a game changer”.

Family dynamics were also affected, particularly in relationships with partners and other children. The stress of caregiving often leads to strain in marriages, with one participant sharing, “My husband has made a couple of comments recently about thinking about what you have to offer someone in a relationship, and I’m thinking well yeah, I probably don’t have much to offer right now, but what do you want from me, I’m like a twenty-four hour a day nurse”. However, some participants also noted that the challenges had strengthened their relationship, as one stated, “My husband and I, our marriage, we were fortunate enough to have a hard thing happen to us…early on in our marriage, having to really lean into each other…so we really had a good, strong foundation, and I think we’re really solid”. Another participant mentioned the ongoing dialog and vulnerability necessary to support one another by stating, “My husband was like, ‘I would never disengage from our marriage, but you are not the person I married.’” Ultimately, the changing needs of the partnership and marriage require ongoing attention to the relationship, as discussed by one participant: “All of the sudden, once the dust settled and after the initial fight or flight wore off, after that first year, and we were still trying to find what the hell life looks like because everything had to change”.

#### 3.1.4. Risk Factors in Families and Healthcare Contexts

The data analysis identified several risk factors that complicated the experience of parents and caregivers. These included the physical distance to healthcare facilities, unhealthy coping mechanisms such as increased alcohol consumption, the impact of other life stressors like bereavement and marital conflict, and the financial strain of balancing work with caregiving responsibility, as well as the necessary role of healthcare provider to their child under extreme circumstances. One participant shared on their commute to the hospital, “It was an hour drive there. It was an hour drive back”.. Related to handling other life events in addition to having a medically complex child, one participant stated, “At the same time, I lost my mom literally two months before I gave birth to him. She was my best, best, best friend. It was the hardest thing I ever had to do”. Participants discussed the adoption of negative coping skills such as using alcohol and over-controlling situations. One participant stated, “My drinking had skyrocketed 18 months after she was born, just dealing with stuff and coping”. Another stated, “I never let anybody but myself or a grandparent drive him anywhere…I became extremely burned out”. When discussing challenges with career and work, one participant shared, “The financial impact of can we be on one income or do we both work and how do we navigate that, and how do you comply with any sort of attendance policy when your life is so insane”.

#### 3.1.5. Protective Factors for Parents and Caregivers

Despite the challenges, participants identified several protective factors contributing to their resilience. Family strength emerged as a key theme, with one participant stating, “Ironically, I will say now we are probably stronger than we ever were”. The support of siblings also played a crucial role, as one participant expressed pride in her other children for their caring and supportive attitudes. Another participant shared, “And he [brother] has just been his protector”. Another participant shared, “And then the other thing that really I’m super proud of are my other two kids and how they are with my oldest daughter, just how loving they are, how caring they are, how considerate they are, how they remind me when I’m being ablest about something or underestimating my daughter in some way”.

Participants also emphasized the importance of self-care and health coping strategies. One participant stated, “I really try to make time at least once a month to do something that I enjoy”. Another stated, “I am pretty diligent about attempting the self-care pieces whenever there is an opportunity to do so”. Another stated, “I feel like our resiliency is that we, for the very most part, try very hard not to blame each other and cause fights”. Appreciating small, meaningful moments was another protective factor, with participants recognizing the value of “quality over quantity” in their family life. One participant expressed, “But when we got that [health] news, we both really took an attitude of quality over quantity”. Concerning making family decisions, one stated, “We are going to make a parenting decision and choose quality of life”. The third participant shared, “Those things are profound joy in a way that they may just have been everyday things that you took for granted before, so I have a great appreciation for that aspect of it as well”.

Finally, participants spoke of a profound sense of empowerment and pride in their ability to manage the complexities of their child’s situations. One participant summarized: “you feel empowered every single day, and you’re proud of yourself every single day, and you’re grieving every single day”.

The phenomenological analysis highlights the complex and multifaceted lived experience of parents and caregivers of children with medical complexity. The interplay between risk and protective factors underscores the resilience of these families despite the profound challenges they face.

## 4. Discussion

Three research questions were posed to explore the lived existential experiences of parents and caregivers of children with medical complexity; findings conveyed themes around lived body, lived time and space, and lived relationships as experiences. In terms of lived body experiences, parents discussed the psychological and physiological effects of their experiences. The experiences of chronic and complex trauma are an important narrative in parent experiences [26,27,31]. Parents have been exposed to extreme medical situations of their own children on multiple occasions and have bore witness to other children’s medical emergencies. Enduring and surviving these experiences has taken a significant toll on wellbeing and mental health, as described by the parents in the study and confirmed in previous studies [27,39,40]. Physiological effects include weight and sleep-related disturbances, anger and temperament issues, ongoing fear, and chronic exhaustion [19,27,39]. Parents’ lived-body experiences also included them dealing with ambiguity, which includes isolation, grief, and death. Parents report feeling lonely and feeling like no one really understands their situation, creating disconnection with others in their lives [40]. Physical, emotional, and social isolation have been reported [7,26,29,39,40]. Grief was a shared experience of the parents in this study; grief related to tangible loss and ambiguous grief related to loss of what could or might have been.

Parents’ lived-time experiences include the impact of their situation on the here and now. Parents report feeling constant exasperation and unpredictability. Other impacts include traveling to and from the city, sacrifice, changes to their lifestyle, and difficulty adjusting to their lives post-children. Many participants reported having difficulty adjusting to their new role as a caregiver and primary healthcare provider of their child. Constant flexibility is required on their part to adapt to ever-changing needs and possible emergencies. The next theme focused on addressing parents’ lived space experiences. Due to their current situation, parents reported being unable to have space and time for themselves. They find it hard to relax and enjoy being a parent. They feel like something bad is always about to happen. Most importantly, parents mentioned not having time for respite and that it is impossible to get away. Parents expressed the inability to engage in life the way that other families can; this includes fully engaging in careers and jobs as well as participating in social activities. They expressed a sense of disappointment, letting others down when they cannot fulfill promises to employers, friends, and family. A theme unique to this group of parents is the chronic and unrelenting requirement of providing home healthcare to their children [16], which impacts overall mental health and trauma symptoms, contributes to burnout, and creates a significant gap in what is possible for parents related to participating in life outside of the home.

The second research question included questions pertaining to the risk factors of these parents and caregivers. Risk factors included the physical distance, travel time, unpredictable nature of their child’s health condition, loss of other family members, pre-existing mental health condition of parents and caregivers, constant comparison to other kids, and unhealthy coping skills. The last research question focused on understanding parents’ and caregivers’ protective factors in their journeys. Many participants reported having personal and familial resilience as one of the biggest protective factors [25]. Furthermore, support from other parents as well as other medical and health professionals was reported to be another protective factor. Some participants also reported the following healthy coping skills and engaging in self-care practices when possible. Many parents saw their family and friends as life-saving connections on whom they can lean for support, despite simultaneously feeling isolation. Families who have had similar experiences were described as a lifeline, and establishing community with other parents is critical to wellbeing [7,40]. A similar theme related to the importance of connection and finding community was found in other studies as participants described the isolation felt among other groups; thus, a shared experience is critical in establishing a community [16]. In addition to their partners, parents revealed how their relationship with their other children has grown stronger over time. Parents revealed pride in how supportive and adaptable their children are with one another. Most importantly, parents reported having an attitude of quality over quantity and the ability to appreciate moments of joy in life.

### Strengths, Limitations, and Future Research

This study illuminates first-hand narrative experiences of parents of children with medical complexity to understand their perceptions of parenting better. A focus group format allowed parents to engage with other parents in similar situations, which is an important resilience factor described in the research findings. The homogeneity of participant characteristics was both a strength and a limitation. The potential similarities of experiences created an opportunity to explore a shared essence; however, diversity in lived experiences is essential in creating transferability outside of the study sample. As a hermeneutic method, the transferability of the results is important; the meaning-making processes of participants are shaped by their diversity; thus, having a heterogenous sample is essential to quality research.

Future studies should include parents from different cultural and economic backgrounds to explore how the intersections of different identities and experiences shape parenting perspectives. The intersection and impact of social determinants of health and other diversity factors on the impact of parenting CMC should be examined. Future research should continue to focus on family process variables that impact the resilience and well-being of families with CMC. As traumatic experiences are prevalent in this group of parents and caregivers, interventions should continue to be explored that are targeted for the relief and management of trauma symptoms to increase family wellness [39,40].

## 5. Conclusions

The current study highlights the voices of 15 parents of CMC and sheds light on the impact of their experiences. Parents caring for CMC have unique demands that ultimately have the potential to significantly alter their mental health, well-being, and experiences of parenting. This study provides implications that can be applied to healthcare settings. Healthcare professionals can continue to provide holistic and trauma-informed care to families with CMC, and parents may benefit from being provided with screenings and psychosocial assessments by medical teams related to depression, trauma symptoms, anxiety, and other mental health issues [11,40]. Furthermore, respite care options such as caregiver options, drop-off day programs, daily or weekly respite programs by community-based agencies, or parent co-op options were suggested by the participants. More research is needed on effective and accessible psychological interventions to support parent mental health and family wellbeing internationally [10]. 

## Figures and Tables

**Table 1:** Demographic Characteristics.

Characteristic		
Race	White	11
	Black	2
	Did not report	2
Gender	Male	1
	Female	14
Household Income	Under $40,000	2
	Between $80–99,000	1
	Over $100,000	10
	Did not report	2
Marital Status	Married	9
	Single/Divorced	4
	Did not report	2

**Table 2:** Theme similarities and differences in the cross-case analysis.

Categories	Theme Similarity and Difference
Lived body (Corporeality)	Significant weight gain Chronic stress Medical trauma Accelerated aging Short temper Exhaustion Fear Poor sleep Isolation Ongoing grief
Lived space (Spatiality)	Difficulty engaging in social activities Restricted mobility Necessity of proximity to healthcare facilities
Lived time (Temporality)	Constantly on edge Lack of respite Inability to relax and enjoy parenting
Lived others (Relationality)	Role conflict (caregiver vs. spouse) Gratitude for the support Shifting family dynamics Reliance on external support
Risk Factors	Distance to healthcare facilities Unhealthy coping mechanisms Additional life stressors Marital strain Financial impact
Protective factors	Family resilience Support Healthy coping strategies Appreciation of small moments A sense of empowerment

## Data Availability

The datasets presented in this article are not publicly available. Requests to access the datasets should be directed to [12].

## References

[1] Caicedo C. (2014). Families with Special Needs Children. J. Am. Psychiatr. Nurses Assoc..

[2] Cohen E. (2011). Children with Medical Complexity: An Emerging Population for Clinical and Research Initiatives. Pediatrics.

[3] National Survey of Children’s Health—Data Resource Center for Child and Adolescent Health [Internet]. https://www.childhealthdata.org/learn-about-the-nsch/NSCH.

[4] Leyenaar J.K., Schaefer A.P., Freyleue S.D., Austin A.M., Simon T.D., Van Cleave J., Moen E.L., O’Malley A.J., Goodman D.C. (2022). Prevalence of Children With Medical Complexity and Associations with Health Care Utilization and In-Hospital Mortality. JAMA Pediatr..

[5] Kuo D.Z., Hall M., Agrawal R., Cohen E., Feudtner C., Goodman D.M., Neff J.M., Berry J.G. (2015). Comparison of Health Care Spending and Utilization among Children with Medicaid Insurance. Pediatrics.

[6] Justin A.Y., Henderson C., Cook S., Ray K. (2020). Family Caregivers of Children with Medical Complexity: Health-Related Quality of Life and Experiences of Care Coordination. Acad. Pediatr..

[7] Bayer N.D., Wang H., Yu J.A., Kuo D.Z., Halterman J.S., Li Y. (2021). A National Mental Health Profile of Parents of Children with Medical Complexity. Pediatrics.

[8] Kuo D.Z., Cohen E., Agrawal R., Berry J.G., Casey P.H. (2011). A National Profile of Caregiver Challenges among More Medically Complex Children with Special Health Care Needs. Arch. Pediatr. Adolesc. Med..

[9] Berry J.G., Hall M., Neff J., Goodman D., Cohen E., Agrawal R., Kuo D., Feudtner C. (2014). Children with Medical Complexity And Medicaid: Spending and Cost Savings. Health Aff..

[10] Harvey A.R., Meehan E., Merrick N., D’Aprano A.L., Cox G.R., Williams K., Gibb S.M., Mountford N.J., Connell T.G., Cohen E. (2024). Comprehensive care programmes for children with medical complexity. Cochrane Database Syst. Rev..

[11] Pilapil M. (2017). Caring for the Caregiver: Supporting Families of Youth with Special Health Care Needs. Curr. Probl. Pediatr. Adolesc. Health Care.

[12] Garner A., Yogman M. (2021). Preventing Childhood Toxic Stress: Partnering with Families and Communities to Promote Relational Health. Pediatrics.

[13] Gilson M., Davis E., Johnson S., Gains J., Reddihough D., Williams K. (2018). Mental health care needs and preferences for mothers of children with a disability. Child Care Health Dev..

[14] Manee F., Ateya Y., Rassafiani M. (2016). A Comparison of the Quality of Life of Arab Mothers of Children with and without Chronic Disabilities. Phys. Occup. Ther. Pediatr..

[15] Thomson J., Shah S.S., Simmons J.M., Sauers H.S., Brunswick S., Hall D., Kahn R.S., Beck A.F. (2017). Financial and Social Hardships in Families of Children with Medical Complexity. J. Pediatr..

[16] Hirt E., Wright A., Kehring A., Wang Y., Torano V., Boles J. (2023). “Fitting the Pieces Together”: The Experiences of Caregivers of Children with Medical Complexity. Hosp. Pediatr..

[17] Cousino M.K., Hazen R.A. (2013). Parenting Stress among Caregivers of Children with Chronic Illness: A Systematic Review. J. Pediatr. Psychol..

[18] Remedios C., Willenberg L., Zordan R., Murphy A., Hessel G., Philip J. (2015). A pre-test and post-test study of the physical and psychological effects of out-of-home respite care on caregivers of children with life-threatening conditions. Palliat. Med..

[19] Cohn L.N., Pechlivanoglou P., Lee Y., Mahant S., Orkin J., Marson A., Cohen E. (2020). Health Outcomes of Parents of Children with Chronic Illness: A Systematic Review and Meta-Analysis. J. Pediatr..

[20] Fisher V., Fraser L., Taylor J. (2023). Experiences of fathers of children with a life-limiting condition: A systematic review and qualitative synthesis. Support. Palliat. Care.

[21] Jaaniste T., Cuganesan A., Ling W., Tan S.C., Coombs S., Heaton M., Cowan S., Aouad P., Potter D., Smith P.L. (2021). Living with a child who has a life-limiting condition: The functioning of well-siblings and parents. Child Care Health Dev..

[22] Jaaniste T., Tan S.C., Aouad P., Trethewie S. (2020). Communication between parents and well-siblings in the context of living with a child with a life-threatening or life-limiting condition. J. Paediatr. Child Health.

[23] von Schulz J., Serrano V., Buchholz M., Natvig C. (2022). Increased behavioral health needs and continued psychosocial stress among children with medical complexity and their families during the COVID-19 pandemic. Infant Ment. Health J..

[24] Mitchell S.M. (2006). Children at Risk for Special Health Care Needs. Pediatrics.

[25] Feinberg M.E., Mogle J.A., Lee J.K., Tornello S.L., Hostetler M.L., Cifelli J.A., Bai S., Hotez E. (2022). Impact of the COVID-19 Pandemic on Parent, Child, and Family Functioning. Fam. Process.

[26] Muscara F., McCarthy M.C., Nicholson J.M., Burke K., Darling S., Rayner M., Anderson V.A. (2018). Trajectories of Posttraumatic Stress Symptoms in Parents of Children with a Serious Childhood Illness or Injury. J. Pediatr. Psychol..

[27] Currie R., Anderson V.A., McCarthy M.C., Burke K., Hearps S.J., Muscara F. (2020). Parental distress in response to childhood medical trauma: A mediation model. J. Health Psychol..

[28] Carmassi C., Dell’Oste V., Foghi C., Bertelloni C.A., Conti E., Calderoni S., Battini R., Dell’Osso L. (2021). Post-Traumatic Stress Reactions in Caregivers of Children and Adolescents/Young Adults with Severe Diseases: A Systematic Review of Risk and Protective Factors. Int. J. Environ. Res. Public Health.

[29] Hall M.F., Hall S.E. (2013). When Treatment Becomes Trauma: Defining, Preventing, and Transforming Medical Trauma. Am. Couns. Assoc..

[30] Sanders M.R., Hall S.L. (2018). Trauma-informed care in the newborn intensive care unit: Promoting safety, security and connectedness. J. Perinatol..

[31] Salley C.G., Axelrad M., Fischer E., Steuer K.B. (2023). But parents need help! Pathways to caregiver mental health care in pediatric hospital settings. Palliat. Support. Care.

[32] Phua D.Y., Kee M.Z., Meaney M.J. (2020). Positive Maternal Mental Health, Parenting, and Child Development. Biol. Psychiatry.

[33] Santiago E.A., Brown C., Mahmoud R., Carlisle J. (2020). Hermeneutic phenomenological human science research method in clinical practice settings: An integrative literature review. Nurse Educ. Pract..

[34] Al-Raisi U., Al Salmi I., Magarey J., Rasmussen P., Hannawi S. (2020). Hermeneutic Phenomenology Research Approach: A Review of the Continuing Professional Development in the Clinical Speciality of Cardiology. Cardiol. Vasc. Res..

[35] Harris P.A., Taylor R., Thielke R., Payne J., Gonzalez N., Conde J.G. (2009). Research electronic data capture (REDCap)—A metadata-driven methodology and workflow process for providing translational research informatics support. J. Biomed. Inform..

[36] Harris P.A., Taylor R., Minor B.L., Elliott V., Fernandez M., O’Neal L., McLeod Delacqua G., Kirby J., Duda S.N. (2019). REDCap Consortium, The REDCap consortium: Building an international community of software partners. J. Biomed. Inform..

[37] Bradbury-Jones C., Sambrook S., Irvine F. (2009). The phenomenological focus group: An oxymoron?. J. Adv. Nurs..

[38] Qualitative Data Analysis Software [Internet]. Delve. https://delvetool.com.

[39] Carter B., Cummings J., Cooper L. (2007). An exploration of best practice in multi-agency working and the experiences of families of children with complex health needs. What works well and what needs to be done to improve practice for the future?. J. Clin. Nurs..

[40] Currie G., Szabo J. (2020). Social isolation and exclusion: The parents’ experience of caring for children with rare neurodevelopmental disorders. J. Qual. Stud. Health Well-Being.

